# Identifying Treatment Modalities for a Multidisciplinary and Blended Care Intervention for Patients With Moderate Medically Unexplained Physical Symptoms: Qualitative Study Among Professionals

**DOI:** 10.2196/12203

**Published:** 2019-04-12

**Authors:** Paula Elisabeth van Westrienen, Martijn F Pisters, Marloes Gerrits, Cindy Veenhof, Niek J de Wit

**Affiliations:** 1 Department of Health Innovation and Technology Fontys University of Applied Sciences Eindhoven Netherlands; 2 Center for Physical Therapy Research and Innovation in Primary Care Utrecht Netherlands; 3 Physical Therapy Research, Department of Rehabilitation, Physical Therapy Science and Sport Brain Center Rudolf Magnus University Medical Center Utrecht Utrecht Netherlands; 4 Department of General Practice Julius Center for Health Sciences and Primary Care University Medical Center Utrecht Utrecht Netherlands; 5 Research Group Innovation of Human Movement Care University of Applied Sciences Utrecht Utrecht Netherlands

**Keywords:** medically unexplained physical symptoms, intervention development, primary care, qualitative study, nominal group technique

## Abstract

**Background:**

Medically unexplained physical symptoms (MUPS) are a substantial health problem in primary care with a high burden for patients, general practitioners, and the health care system. Most studies focus on chronic MUPS patients. Little research is conducted in patients with moderate MUPS, and an effective primary care intervention for prevention of chronic MUPS is lacking.

**Objective:**

The objective of our study was to identify treatment modalities based on expert opinions for the development of a multidisciplinary and blended intervention for patients with moderate MUPS to prevent chronicity.

**Methods:**

Two focus groups with 8 and 6 experts (general practitioners, physical therapists, psychologists, and mental health nurses) were carried out. The focus groups were structured using the nominal group technique.

**Results:**

A total of 70 ideas were generated from two nominal group meetings, and 37 of these got votes, were included in the rank order, and were sorted into 8 separate themes. According to the participants, the most important treatment modalities for a multidisciplinary and blended intervention in patients with moderate MUPS were (1) coaching to a healthier lifestyle, (2) education regarding psychosocial factors, (3) therapeutic neuroscience education, (4) multidisciplinary intake, (5) multidisciplinary cooperation and coordination, (6) relaxation or body awareness exercises, (7) clear communication by professionals to the patient, and (8) graded activity. Five independent researchers checked the ideas and linked them to themes to confirm the content analysis and check the validity of the themes.

**Conclusions:**

From professional expert perspectives, 8 themes should be included in a multidisciplinary and blended intervention to prevent chronicity. These themes provide a first step in developing an intervention for patients with moderate MUPS. Future research should focus on further development steps in which patients with moderate MUPS should be involved to determine if the intervention matches their needs.

## Introduction

Medically unexplained physical symptoms (MUPS) are physical complaints (eg, pain, fatigue, dizziness) that last for at least a few weeks and cannot be explained by a medical condition after adequate medical examination [1,2]. Approximately 20% of patients with MUPS still experience unexplained physical symptoms after 3 months, and a third of patients presenting with MUPS maintain unexplained symptoms after 5 years [3]. Symptoms can be categorized into moderate MUPS and chronic MUPS [2,4]. Moderate MUPS symptomatology can be of any type and intensity in 2 or 3 domains (eg, musculoskeletal, fatigue, cardiology-respiratory) with psychological and physical distress. Chronic MUPS symptomatology is within more domains with psychological and physical dysfunction (eg, fibromyalgia, chronic fatigue syndrome, irritable bowel syndrome) [2,4]. The estimated prevalence of moderate MUPS is 15%, and chronic MUPS occurs in approximately 2.5% of patients in primary care [4-6]. The burden (eg, physical, social, emotional) of MUPS is high based on the decrease in quality of life and increase in health care use for patients [7-9]. Furthermore, the burden is high for general practitioners and society since general practitioners do not recognize patients with MUPS early, and they experience difficulties in treating and managing patients with MUPS [10-12], leading to increased direct health care costs and indirect costs (eg, work- and insurance-related costs) [9].

Many studies have already been conducted in patients with chronic MUPS to assess the efficacy of psychological, pharmacological, exercise therapy, or combined treatment approaches [13-18]. So far, systematic reviews based on low-quality evidence suggest that cognitive behavioral therapy might be an effective psychological treatment [17,18]. The focus of pharmacological interventions should be on action of the central nervous system (eg, antidepressants) instead of restoration of peripheral physiological dysfunction (eg, nonsteroidal anti-inflammatory drugs) [16]. Furthermore, compelling evidence for neuroscience education is found on pain, disability, catastrophization, and physical performance [13]. In a session on neuroscience education, the patient is educated on the neurobiology and neurophysiology of pain and pain processing by the nervous system [13]. Systematic reviews based on low- to moderate-quality evidence suggest that exercise therapy has a positive effect on physical function [14,15]. Despite the evidence for the more isolated interventions, it is suggested that treatments should be multimodal in patients with MUPS, with components of exercise, education, and integrating aspects of a psychological approach [13,16,17].

For the development of multimodal interventions, expert opinions and patient needs should be taken into account [19]. Different studies have already focused on the management of MUPS. In a qualitative analysis on expert opinions, some relevant elements were identified for successful management of MUPS: creating a safe therapeutic environment and using generic (eg, motivational interviewing) and specific (eg, cognitive approaches) interventions [20]. Furthermore, earlier research has indicated that explanation of the symptoms is an important management strategy in patients with MUPS [21,22].

Many qualitative and quantitative studies have focused on patients with chronic MUPS [13-18,21,23-26]. Little research has been conducted in patients with moderate MUPS, but preventing chronicity in moderate MUPS to decrease the burden for patients, general practitioners, and society is important [27]. Recently, we developed a screening method (PRESUME: preventive screening of medically unexplained physical symptoms) to identify patients with moderate MUPS using the electronic medical records of the general practitioner. The method consists of 3 steps based on consultation frequency, exclusion of medical and/or psychiatric diagnosis, and identification of chronic MUPS and moderate MUPS. Patients are identified with chronic MUPS when they are diagnosed with a functional somatic syndrome (eg, fibromyalgia, chronic fatigue syndrome, or irritable bowel syndrome), and patients with moderate MUPS have MUPS-related symptoms without a MUPS diagnosis. Despite its limited prognostic accuracy, the PRESUME screening method facilitates identification of patients with moderate MUPS. In the next step we aim to develop an effective multidisciplinary and blended primary care intervention to prevent chronicity in patients with moderate MUPS. The expectation is that the integration of face-to-face sessions with eHealth modules, called blended care, will promote self-management. Furthermore, a blended care intervention may lead to a decrease of costs since the face-to-face sessions are not performed on a weekly basis. Blended care has already been proven effective in other studies [28,29]. The intervention will be performed in primary care; therefore, a physical therapist and mental health nurse should be involved in the intervention since both disciplines treat patients with MUPS in primary care in the Netherlands [2]. For development of the intervention, the Medical Research Council (MRC) framework will be used. The MRC framework include several phases: development, feasibility and piloting, evaluation, and implementation [19]. In this study we focused on identifying existing relevant themes for the intervention as a first part of the development phase of the MRC framework. Professionals involved in the clinical management of MUPS were asked to participate. The aim of this study was to identify expert-based treatment modalities for a multidisciplinary intervention for patients with moderate MUPS in primary care.

## Methods

### Design

A qualitative study using focus groups according to the nominal group technique (NGT) was performed [30]. Preconditions were that the intervention will be multidisciplinary and blended, with the focus on self-management.

### Participants

Professionals involved in the clinical management of patients with MUPS were approached to participate in the study. Eligible participants were selected through purposive sampling and finally included based on availability. Purposive sampling was applied to obtain variation in disciplines (general practitioner, psychosomatic physical therapists, health care psychologists, and mental health nurses). The number of participants in a nominal group meeting was based on the recommendation of a maximum of 9 or 10 participants per group [31]. Based on the involvement of different disciplines, multiple nominal group meetings were organized [32]. We started with organizing 2 nominal group meetings, so the participants were divided into 2 groups. If there were no agreement between the items mentioned in the first 2 meetings, extra meetings with other participants would be organized until data saturation was achieved. The study was carried out according to Dutch privacy legislation rules. Written informed consent was obtained from all participants before the start of the focus group. In the first focus group, one general practitioner, one physical therapist, 2 psychosomatic physical therapists, 2 health care psychologists, and 2 mental health nurses participated. In the second focus group, 2 general practitioners, one physical therapist, 2 psychosomatic physical therapists, and one psychologist/physical therapist participated. Since the results of the second group discussions did not add major new ideas compared to the ideas identified in the first group, saturation was assumed and no additional group sessions were held.

Participants had a median work experience of 18 years (interquartile range [IQR] 20), where 21% (3/14) had less than 10 years of work experience, 29% (4/14) had 10 to 20 years of working experience, and 50% (7/14) had 20 years or more of work experience. In addition, participants had a median experience of treating patients with MUPS of 9 years (IQR 18), where 50% (7/14) had less than 10 years of experience in treating patients with MUPS, 21% (3/14) had 10 to 20 years of experience in treating patients with MUPS, and 29% (4/14) had 20 years or more experience in treating patients with MUPS. Some participants had other work activities besides their profession such as a researcher, teacher, or public administrator. Demographic characteristics of the participants are shown in Table 1.

**Table 1:** Demographic characteristics of the participants (n=14).

Characteristics		Values
**Profession, n (%)**		
	General practitioner	3 (21)
	Physical therapist	2 (14)
	Psychosomatic physical therapist	4 (29)
	Psychologist/physical therapist	1 (7)
	Health care psychologist	2 (14)
	Mental health nurse	2 (14)
Female, n (%)		9 (64)
Age in years, median (IQR^a^)		46.5 (20)
Years of general work experience, median (IQR)		18 (20)
Years of experience treating patients with MUPS^b^, median (IQR)		9 (18)
**Other work activities^c^, n (%)**		
	Research assistant	1 (7)
	Junior researcher	1 (7)
	Postdoctorate researcher	1 (7)
	Senior researcher	1 (7)
	Clinical health scientist	1 (7)
	Clinical epidemiologist	1 (7)
	Teacher	5 (36)
	General practitioner, special interest musculoskeletal	1 (7)
	General practitioner, special interest mental health care	1 (7)
	Public administrator	1 (7)

^a^IQR: interquartile range.

^b^MUPS: medically unexplained physical symptoms.

^c^Some participants are classified in multiple categories.

### Procedure

NGT is a formal stepwise consensus procedure that uses structured interaction within the group. Ideas were generated focusing on optimization of moderate MUPS management [30]. NGT is a structured group meeting, which is of interest in a heterogenic group of participants [33]. This technique enables participants to gather individual ideas, obtain ideas from other members, and rank ideas with equal input from all participants.

- Introduction of the nominal question: welcome and introduction of NGT and the nominal question.
- Silent generation of ideas: participants are asked to write down their individual list of ideas that come to mind regarding the nominal question without discussing with or consulting others.
- Presenting of ideas: sheets with individual ideas are gathered, and each participant presents their ideas to the group without discussion.
- Group discussion: all ideas are evaluated, clarified, and discussed one by one. Ideas can be specified when necessary. Similar items can be merged but only after agreement of all participants.
- Voting and ranking of ideas: participants are asked to individually rank the 5 most important items without discussion with other group members. Scores are summed (an item receives 5 points for a number 1 position, 4 points for a number 2 position, etc), and a final rank order is presented.

A week before the meeting, participants received information about the meeting, and the research question was introduced by the principal researcher: “Which treatment modalities should be part of the multidisciplinary treatment program for moderate MUPS to prevent chronic MUPS?” At the beginning of the meeting, participants were introduced and the role of the facilitator (assistant; presents all ideas and rankings in PowerPoint) and principal researcher (moderator of the discussion) were explained. In addition, the purpose and procedure of the meeting were explained, the research question was displayed, and the definition of moderate MUPS was specified (patients who have had at least 5 general practice consultations during the past 12 months of which at least 3 were based on the presence of MUPS-related symptoms; furthermore, patients should have psychological and physical distress). Subsequently, the 4 structured steps according to the NGT procedure were explained and followed [30].

The first step is silent generation, where all participants wrote down ideas around the question individually and privately for approximately 20 minutes. The second step was a round-robin format, where all participants shared their ideas one by one with the group. One participant at the time stated a single idea, which was presented on a screen in front of the group by the facilitator. This process was continued until all ideas from participants were listed and displayed on the screen. There was no discussion at this stage. In the third step, all collected ideas were clarified and discussed in the group. Similar ideas were grouped together but only after agreement by all participants. Discussion ended when no new ideas were generated or grouped together and data saturation within the group was thus achieved. In the fourth step, participants were able to independently rank 5 ideas from all generated ideas. In this ranking process, participants gave 5 votes to the most important idea and the fifth most important idea got one vote. After the 4 steps, the facilitator collected the voting sheets, and the scores for each idea were presented. The group meeting was audiotaped to verify data and use the information for ongoing analysis after the meeting.

### Data Analysis

NGT enables participants to be involved in data analysis by composing a rank order. Rank orders of the 2 groups were merged into one final rank order using a structured method for analyzing multiple group data [34]. All ideas were listed in the final rank order to combine ideas into themes by the principal researcher (content analysis). Subsequently, each theme got a definition. To confirm the content analysis as well as increase the reliability, 5 independent researchers who were not involved in the study checked the ideas and decided to which theme they belonged to determine if themes should be more clearly defined or maybe combined or redivided [34]. In the last step, all themes were ranked according to the number of ideas that formed the theme, the number of times the ideas were ranked in the top 5, and the relative score of the ideas within the group rankings. Only the ideas that had received votes were included in the rank order. Multigroup data analysis procedure [34] is as follows:

- Capture data on computer: sets of items with the individual and group scores for each item can be entered on a spreadsheet.
- Identifying the overall top 5 per group: sets of items were ordered according to the importance of the items as scored by each group. Subsequently, the top 5 of the most important items of each group are identified as described by the steps of van Breda et al [34].
- Content analysis of the data: the principal investigator (EvW) will combine the items from all groups into groups of items. This process is repeated a few times, and themes are created. An item can fall into one theme only. Subsequently, a definition to each theme is created. This is a time-consuming process.
- Confirm the content analysis: the content analysis is peer-reviewed by independent researchers who have not been involved in the NGT research process. Subsequently, the principal investigator determines whether themes should be more clearly defined or maybe combined or redivided.
- Calculating combined ranks: the relative importance of each theme to all the groups combined is calculated. The final rank provides a consolidation of all items generated and ranked by the participants.

## Results

From the 2 nominal group meetings, 70 ideas were generated (37 in group 1 and 33 in group 2), of which 37 received scores from the participants (19 in group 1 and 18 in group 2). All ideas from both focus groups were ordered according to the scores of the participants. Subsequently, the top 5 ideas were identified. The idea with the highest score in both focus groups was “education about the complaints of the patient.” Both focus groups indicated that it should be at the start of an intervention. Participants of the first focus group scored as the second most important idea “education about factors which affect the complaints, to make the connection with possible perpetuating factors.” The third most important idea was the “treatment demand.” The participants found it important that the treatment demand should be clear at the end of the intake for a tailored intervention. The fourth most important idea was that professionals should pay attention to patients’ lifestyle, where self-management in general daily life of patients is of interest. The fifth most important idea of the first focus group was that “patients should get more insight in their emotions, behavior, and thoughts in relation with the complaints.” In the second focus group, the participants scored as second most important idea “interdisciplinary collaboration.” The third most important idea was “the patient should have problem-solving skills.” Participants expected that patients with problem-solving skills would recognize challenges as well as develop self-management strategies. Fourth, “cognitive behavioral interventions are of interest to help patients managing their problems.” The participants found it important that patients learn to make the connection between their thoughts, feelings, and behavior and their complaints with cognitive behavioral interventions. Finally, participants in the second focus group mentioned “education and coaching on lifestyle” as the fifth most important idea.

After the identification of ideas and their ranking in both focus groups, the ranked ideas were merged into one final rank order according to the structured method for analyzing multiple group data [34]. This final rank order was analyzed, and all ideas were divided into themes by the principal researcher (Multimedia Appendix 1). Eight separate themes with definitions were composed by the principal researcher.

1. Coaching to a healthier lifestyle: coaching to a healthier lifestyle and behavioral changes through self-management as well as a balance between burden and capacity with attention to coping strategies
2. Education regarding psychosocial factors: education on possible precipitating and maintaining factors of the complaints with the connection between thoughts, emotions, and behavior
3. Therapeutic neuroscience education: education of central sensitization
4. Multidisciplinary intake: a multidisciplinary intake with both physical aspects (eg, by the physical therapist) and mental aspects (eg, by a mental health nurse). During the intake, both disciplines should focus on the complaints (also checking if the patient has doubts about having a medical diagnosis), cognitions, emotions, behavior, and social environment of the patients, but from a different perspective. Additionally, the treatment demand and goals of the patient should be clear before the actual start of the intervention
5. Multidisciplinary cooperation and coordination: multidisciplinary cooperation between, for example, the general practitioner, physical therapist, and mental health nurse with established consultation meetings where the general practitioner will have the coordinating role during the intervention
6. Relaxation or body awareness exercises: relaxation or body awareness exercises should be part of the intervention (eg, general relaxation techniques [progressive relaxation or autogenic training], mindfulness, and exercises according to psychomotor therapy)
7. Clear communication of professionals to the patient: professionals should express themselves in the same way toward the patient during education sessions and should have insight into their own cognitions about MUPS
8. Graded activity: gradually increasing the amount of physical activity in a time-contingent way based on individual goal setting, using preset quotas and principles of operant conditioning

The composed themes were validated by 5 independent researchers who were not involved in the study. They checked the ideas and decided in which theme they belong. This led to the adjustment of 7 ideas into other themes and a more clear definition of 3 themes. After validating our composed themes, the relative importance of each theme was determined according to a ranking score. “Coaching to a healthier lifestyle” had the highest ranking score and was the first theme. “Graded activity” had the lowest ranking score. This ranking score indicated which parts of the multidisciplinary and blended primary care intervention were most important from a professional expert perspective.

## Discussion

### Principal Findings

The aim of this study was to determine treatment modalities according to professional experts for the development of a multidisciplinary and blended primary care intervention in patients with moderate MUPS to prevent chronic MUPS. According to the ideas and their ranking, 8 themes were important. Our study is the first qualitative study focusing on patients with moderate MUPS, since earlier research focused on patients with chronic MUPS [20,21]. Additionally, qualitative studies on MUPS and health care professionals included general practitioners only. As far as we know this is the first qualitative study in which all health care professionals involved in management of MUPS in primary care were included.

Although comparison with results of earlier research is difficult since it focused on management of chronic MUPS, some of the themes we created in our study were also proven effective in patients with chronic MUPS [2,13,35,36]. The Dutch Multidisciplinary Guideline for MUPS and Somatoform Disorders advises to start the intake by exploring the somatic, cognitive, emotional, behavioral, and social dimensions of the complaints [2], which is in line with the results of the nominal group meetings. Evidence for neuroscience education is found for patients with chronic musculoskeletal pain disorders [13]. Furthermore, progressive muscle relaxation as a relaxation exercise is effective on intensity and number of symptoms, quality of life, and comorbid symptoms for patients with multiple somatoform symptoms [36], and graded activity had a medium effect for patients with chronic fatigue syndrome on fatigue severity reduction [35]. These similarities could possibly be due to the fact that professional experts might know the effective interventions for patients with chronic MUPS and found them also applicable for patients with moderate MUPS. It can also be related to the clinical presentation, as both patients with moderate MUPS and patients with chronic MUPS experience physical and psychological problems [4]. Although the themes partly overlap with the key management aspects of chronic MUPS, none of the intervention studies on patients with chronic MUPS integrated all aspects in a multidisciplinary and blended primary care intervention.

### Strengths and Limitations

This study has a few strengths. First, a group of experts with representatives in all relevant disciplines was included. Therefore, an answer as broad as possible to our question was gathered. Second is the choice of the nominal group technique. Since the intervention for patients with moderate MUPS will be multidisciplinary, we provided a qualitative study with a heterogenic group of professional experts. The structure of the NGT enables group discussion and assures equal input from all participants instead of the possibility that only one participant is mostly speaking [37]. A third strength is that we checked our content analysis by letting independent researchers who were not previously involved in the study check the ideas and decide in which theme they belong [34]. This step in the data analysis of the study is to test the content validity of our themes and enhances the interrater reliability. Beside the strengths, some limitations should be noted. Firstly, a limitation of the purposive sampling strategy is the nonrandom selection of participants. However, with this nonrandom selection of participants, a representative group of all relevant disciplines involved in the clinical management of patients with MUPS was gathered. Furthermore, all participants were aware of the last scientific findings in MUPS research. If a random selection of participants were conducted, the possibility existed that not all relevant disciplines would be selected. Second, some participants seemed to have difficulties with the focus on patients with moderate MUPS as a target group and therefore mentioned ideas that had probably more focus on chronic MUPS. At the beginning of the meeting, the principal researcher pointed out the definition of moderate MUPS and specified to mention ideas that focused on treatment modalities for patients with moderate MUPS. The participants got the definition of moderate MUPS on paper. During the discussion step of the NGT, participants addressed to each other that some ideas might better fit as treatment modalities for patients with chronic MUPS. This led to the removal of some ideas by participants but only after agreement of all participants. In this way, ideas with the focus on treatment modalities for patients with moderate MUPS remained and could get ranked during the last step of the NGT procedure. A third limitation is that generalizability to foreign countries might be complex due to the differences with respect to the health care systems of other countries. Despite these differences, our identified themes for an intervention can be applied in other health care systems or countries since the context for an intervention will not differ.

The results of this study are the basis for the development of a multidisciplinary and blended primary care–focused intervention for patients with moderate MUPS to prevent chronicity. A new primary care intervention would be of great value in clinical practice. In the next step, principles of the Center for eHealth Research road map can be used to focus on the integration of face-to-face sessions using the eHealth modules [38].

### Conclusion

From professional expert perspectives, 8 themes should be included in a multidisciplinary and blended intervention to prevent chronicity. These themes provide a first step in developing an intervention for patients with moderate MUPS. Future research should focus on further development steps of the MRC framework in which patients with moderate MUPS should be involved to determine if the intervention matches their needs.

## Appendix

Multimedia Appendix 1 All ideas from both focus groups divided into eight themes.

## Abbreviations

IQR: interquartile range
MRC: Medical Research Council
MUPS: medically unexplained physical symptoms
NGT: nominal group technique
PRESUME: preventive screening of medically unexplained physical symptoms
