# Supplementary material for: Identifying Treatment Modalities for a Multidisciplinary and Blended Care Intervention for Patients With Moderate Medically Unexplained Physical Symptoms: Qualitative Study Among Professionals
Source: JMIR Ment Health. 2019 Apr 12;6(4):e12203. doi: 10.2196/12203 (PMC6484260; doi:10.2196/12203)
Supplement: Multimedia Appendix 1 [file mental_v6i4e12203_app1.pdf]

Multimedia Appendix 1. All ideas from both focus groups divided into eight themes.

Themes	Items focus group 1	Items focus group 2
1. Coaching to a healthier lifestyle	- Coaching on lifestyle<br>- Balance between performance and capacity<br>- Adjustment of the coping style<br>- Communication coaching techniques (motivational interviewing, problem solving)	- Acceptance and commitment therapy<br>- Provocative psychology<br>- The patient should develop problem-solving skills<br>- Coaching towards a daily schedule<br>- Lifestyle education and coaching<br>Involvement of relatives in the intervention
2. Education regarding perpetuating factors	- Psycho-education about perpetuating factors<br>- Insight in persisting complaints with the connection between thoughts, emotions and behaviour<br>- Insight in the link between the complaints and the family system	- Cognitive behavioural interventions
3. Therapeutic neuroscience education	- Psycho-education about MUPS	- Education about MUPS with reference to an explanatory model
4. Multidisciplinary intake	- Treatment demand<br>- Identifying complaints according to the SCEGS (somatic, cognitive, emotional, behavioural and social factors)<br>- Checking reassurance regarding the absence of a medical diagnosis<br>- Symptom registration in a patient diary	- Identifying illness beliefs<br>- Monitoring physical behaviour<br>- Creating and evaluating an action plan
5. Multidisciplinary cooperation and coordination	- Regular multidisciplinary consultations	- Interdisciplinary collaboration
6. Relaxation / body awareness exercises	- Body awareness<br>- Relaxation exercises<br>- Coping with anxiety/stress<br>- Emotion regulation through movement	- Relaxation techniques<br>- Body awareness exercises<br>- Psychomotor interventions
7. Clear communication of professionals to the patient	- Clear referral of the general practitioner	- Training of the professional (communication techniques)<br>- Training of the professionals according to their cognitions about MUPS
8. Graded activity	- Graded activity	- Time contingent approach
